# Editorial: Revolutionizing cancer care: the promise of liquid biopsy assays for healthcare equity

**DOI:** 10.3389/fonc.2024.1493833

**Published:** 2024-10-18

**Authors:** Chad Brenner, Carolyn Y. Fang

**Affiliations:** ^1^ Department of Otolaryngology Head & Neck Surgery, Ann Arbor, MI, United States; ^2^ Department of Pharmacology, Ann Arbor, MI, United States; ^3^ Cancer Prevention and Control Program, Fox Chase Cancer Center, Philadelphia, PA, United States

**Keywords:** CtDNA, multicancer early detection, HPV - human papillomavirus, liquid biopsy, cancer disparities, healthcare equity, rural medicine

Due to the growth of the aging population in the United States, it is predicted that the annual number of cancer cases will increase 49% in the next 25 years (1). Although advances in cancer screening and treatment have improved outcomes and reduced cancer mortality, these advances have not benefited all populations equally. Disparities in cancer risk and outcomes continue to be observed across race, ethnicity, socioeconomic status, and geography (2). Numerous factors including access barriers, time, costs, or insufficient personnel, equipment, and available appointments contribute to persistent disparities and have been difficult to overcome. Emerging tools, such as liquid biopsy assays, are being explored for their potential utility in addressing barriers to screening and disease surveillance. Liquid biopsy assays represent a broad category of diagnostic tests that analyze genetic material, proteins, or other biomarkers found in bodily fluids (e.g., blood, urine, or saliva) in order to detect or monitor for various diseases including cancer. In this Research Topic entitled “Revolutionizing Cancer Care: The Promise of Liquid Biopsy Assays for Healthcare Equity,” we have assembled a series of papers in which authors consider the key issues surrounding liquid biopsy tests and offer insights on their potential impact for patient care, healthcare economics and cancer disparities.

Breast cancer is the most commonly diagnosed cancer among women worldwide (3). Mansour et al. reflect on the potential role of liquid biopsy for the early detection and monitoring of breast cancers and in addressing the global burden of breast cancer. While mammography remains the most widely used screening tool, the authors highlight the potential of emerging blood-based tests to offer a non-invasive, accessible, and potentially more accurate alternative. However, the authors acknowledge that the development and integration of these tests into clinical practice will require significant investment, collaboration with regulatory bodies, and endorsements from reputable organizations to ensure they meet quality and safety standards. Despite these challenges, the authors point to the rapidly expanding market for *in vitro* diagnostic testing for breast cancer, particularly in regions with high incidence and mortality rates, which presents a substantial economic opportunity. Beyond the economic implications, the widespread adoption of these innovative tests could revolutionize breast cancer screening and diagnosis, ultimately improving quality of life and saving lives on a global scale.

The potential of HPV circulating tumor DNA (ctDNA) to serve as a biomarker for managing HPV-positive oropharyngeal cancer (HPV-OPC), particularly in rural populations facing significant barriers to cancer care, is examined in the paper by Windon and Haring. While the data supporting HPV ctDNA’s sensitivity in detecting recurrence is favorable, the authors underscore the need for further research to optimize its clinical use. Challenges such as the need for reliable methods to confirm recurrence, the occasional absence of detectable ctDNA in some patients, and the reliance on conventional surveillance methods in cases of false negatives, are crucial issues that remain to be addressed. Despite these limitations, the development of HPV ctDNA assays represents a significant advancement, offering the potential to improve access to care through less invasive diagnostic and surveillance methods. This is particularly impactful for rural populations, where such advancements could enhance cancer-related health equity by making comprehensive surveillance more accessible and potentially reducing the need for frequent in-person exams and imaging.

The remaining 2 papers focus on multi-cancer early detection (MCED) tests, which are an area of active research for many programs. While standard cancer screening methods have been effective in reducing morbidity and mortality for certain cancers, their reach and effectiveness are limited, especially among underserved populations. MCED tests offer a promising alternative, capable of detecting a broader range of cancers with a simple blood test, which could significantly improve adherence to screening. At present, a number of clinical trials assessing the efficacy and safety of MCED tests are underway, and the paper by Thompson et al. highlights key steps that should be taken to ensure that MCED clinical trials successfully engage and include participants from diverse populations. Their paper emphasizes the importance of equitable distribution and representation in clinical trials, advocating for the inclusion of diverse populations in research to ensure that the benefits of these emerging technologies are accessible to all. It also highlights the potential of community health workers (CHWs) and health system learning communities as pivotal strategies for engaging underrepresented groups in MCED trials, thereby contributing to the broader goal of reducing cancer disparities and improving public health outcomes.

Thompson and Baskin expand these ideas further, by discussing how multi-cancer early detection (MCED) tests can fulfill their potential to reduce cancer disparities without exacerbating existing inequalities. The authors emphasize the importance of including diverse populations in clinical trials to ensure that MCED tests are effective across all demographic groups, particularly those who are most affected by cancer disparities. They also highlight key challenges, such as the affordability of MCED tests, the need to build trust in marginalized communities, and the importance of high-test specificity to avoid unnecessary follow-ups. The paper underscores the necessity of patient navigation programs to guide individuals through the healthcare system, especially in underserved populations. By addressing these complex factors early in the development and implementation of MCED tests, the authors argue that these tools can be leveraged to close gaps in cancer screening and improve outcomes for historically marginalized groups. Their call for further research and careful implementation is crucial for ensuring that the benefits of MCED testing are equitably distributed, ultimately contributing to a more just and effective cancer care system.

The papers in this Research Topic highlight the potential of emerging liquid biopsy tests to improve cancer care, particularly among underserved populations. There is consensus regarding the need for equitable access, inclusion of diverse populations in clinical trials, and the development of affordable, sensitive tests to reduce cancer disparities. Importantly, this Research Topic underscores how these innovations, especially in rural and high-incidence regions, could transform cancer detection and lead to improved patient outcomes.
